# Supplementary material for: Self-Healing and Highly Stretchable Hydrogel for Interfacial Compatible Flexible Paper-Based Micro-Supercapacitor
Source: Materials (Basel). 2021 Apr 8;14(8):1852. doi: 10.3390/ma14081852 (PMC8070428; doi:10.3390/ma14081852)
Supplement: Supplementary file 1 [file materials-14-01852-s001.zip › materials-1153525-supplementary-send to publish.docx]

*Supplementary Materials*

Article

Self-Healing and Highly Stretchable Hydrogel for Interfacial Compatible Flexible Paper-Based Micro-Supercapacitor

Yutian Wang ^1^, Yunhui Shi ^1^, Yifan Gu ^1^, Pan Xue ^1^ and Xinhua Xu ^1,2,^*

**Citation:** Wang, Y.; Shi, Y.; Gu, Y.; Xue, P.; Xu, X. Self-Healing and Highly Stretchable Hydrogel for
Interfacial Compatible Flexible Paper-Based Micro-Supercapacitor. *Materials* **2021**, *14*, x. https://doi.org/10.3390/xxxxx

Academic editor: Daisuke Tashima

Received: 5 March 2021

Accepted: 5 April 2021

Published: date

**Publisher’s Note:** MDPI stays neutral with regard to jurisdictional claims in published maps and institutional affiliations.

**Copyright:** © 2021 by the authors. Submitted for possible open access publication under the terms and conditions of the Creative Commons Attribution (CC BY) license (http://creativecommons.org/licenses/by/4.0/).

^1^	School of Materials Science and Engineering, Tianjin University, Tianjin 300072, China; [15022345024@163.com](mailto:15022345024@163.com) (Y.W.); [yhshi@hebut.edu.cn](mailto:yhshi@hebut.edu.cn) (Y.S.); [gyf18822066907@163.com](mailto:gyf18822066907@163.com) (Y.G.); xp784422160@163.com (P.X.)

^2^	Tianjin Key Laboratory of Composite and Functional Materials, Tianjin University, Tianjin 300072, China

*****	Correspondence: xhxu@tju.edu.cn; Tel.: +86-22-27406127

**Table S1.** Concentration of PVA and Pluronic in prepared hydrogels.

| **Hydrogels** | **PVA (g)** | **Pluronic (g)** | |  |
| --- | --- | --- | --- | --- |
| BPVA-Pluronic-3 | 2.1 | | 1.1 | |
| BPVA-Pluronic-5 | 1.6 | | 1.6 | |
| BPVA-Pluronic-6 | 1.1 | | 2.1 | |

**Figure S1.** EDX spectrum of C (b), O (c), B (d), K (e) and Cl (f) of BPVA-Pluronic-3 hydrogel section (a).

**Figure S2.** Stress−strain curves of BPVA-Pluronic-5 hydrogels at various tensile speed.

**Figure S3.** The stress−strain curves of the original pure PVA hydrogel and cut-healed pure PVA hydrogel for healing times of 6 h.

**Figure S4.** (**a**) The frequency dependent impedance plots of the BPVA-Pluronic hydrogel electrolytes with various concentration of KCl. (**b**) The frequency dependent impedance plots of the BPVA-Pluronic hydrogel electrolytes with varied PVA/Pluronic ratio.

**Table S2.** Ionic conductivity of various BPVA-Pluronic electrolyte.

| Hydrogels | Pluronic/PVA ratio | KCl concentration | Ionic conductivity |
| --- | --- | --- | --- |
| BPVA-Pluronic-5 | 1 | 1 mol/L | 27.57 mS cm^-1^ |
| - | 1 | 0.5 mol/L | 14.65 mS cm^-1^ |
| - | 1 | 1.5 mol/L | 5.68 mS cm^-1^ |
| - | 1 | 2 mol/L | 4.57 mS cm^-1^ |
| BPVA-Pluronic-3 | 0.33 | 1 mol/L | 6.98 mS cm^-1^ |
| BPVA-Pluronic-6 | 0.67 | 1 mol/L | 24.54 mS cm^-1^ |

**Figure S5.** Rheological properties of the MWCNT dispersion electrode ink. (**a**) Functional relationship between apparent viscosity and shear rate and (**b**) Storage modulus G’ (black curve) and loss modulus G’’ (red curve) as the function of shear stress.

**Figure S6.** (**a**) The 3D structure of the interface between the edge of MWCNT interdigitated microelectrodes and cellulose paper substrate. (**b**) The 3D structure of a single MWCNT interdigitated microelectrode.

**Figure S7.** (**a**) The cross-section SEM image of the interface between MWCNT interdigitated microelectrodes and cellulose paper substrate. (**b**) The cross-section SEM image of the interface between BPVA-Pluronic hydrogel electrolyte and MWCNT interdigitated microelectrodes.

**Figure S8.** Potential-specific capacity curves of the MSC at various current densities.

**Figure S9.** Self-discharge rate curve of the MSC.

**Table S3.** A detailed comparison of specific capacitance of various carbon-based MSCs.

| **Substrate** | **Electrode** | **Electrolyte** | **Specific capacitance (mF cm^-2^)** | **Reference** |
| --- | --- | --- | --- | --- |
| Si | Graphene | PSSH | 0.7 | [40] |
| PG paper | Graphene | PVA/H_3_PO_4_ | 5.296 | [41] |
| PI membrane | Graphene | PVA/H_3_PO_4_ | 0.62 | [42] |
| PET-PSA | Graphene/PANI | PVA/ H_2_SO_4_ | 3.8 | [43] |
| Plastic | Graphene | PS-PMMA-PS/ [EMIM][TFSI] | 0.268 | [45] |
| Kapton | Graphene | Nano-graphene oxide | 0.313 | [47] |
| Si | TiC/CDC | PC/(NEt_4_BF_4_) | 1.5 | [48] |
| Textile | Graphene/AU | PVA/H_3_PO_4_ | 0.76 | [49] |
| PET | Graphene | PVA/LiCl | 3.9 | [50] |
| Carbon microfibers | MWCNT | PVA/H_3_PO_4_ | 5.1 | [54] |
| Cellulose paper | MWCNT | PVA/KCl | 6.42 | This work |

**Figure S10.** (**a**) The bending performance of the MSC. (**b**) The self-healing efficiency of the MSC. (**c**) The cycling performance of the MSC at the current density of 300 μA cm^-2^.

**Video S1**. A movie exhibiting the high self-healing efficiency of the MSC.
